# Six artificial intelligence innovation strategies applied to autism spectrum disorder research: A narrative review

**DOI:** 10.1002/ped4.70038

**Published:** 2026-01-27

**Authors:** Ting Zhang, Zhenkun Cao, Wei Li, Zhihai Lv

**Affiliations:** ^1^ Department of Rehabilitation Medicine and Physiotherapy School of Rehabilitation Medicine, Jiamusi University Jiamusi Heilongjiang China; ^2^ Department of Pediatric Rehabilitation Longgang District Maternity & Child Healthcare Hospital of Shenzhen City (Affiliated Shenzhen Women and Children's Hospital (Longgang) of Shantou University Medical College) Shenzhen Guangdong China; ^3^ Department of Sports Sciences Faculty of Social Sciences Georg‐August University of Göttingen Göttingen Germany

**Keywords:** Artificial intelligence, Autism spectrum disorder, Digital twin, Federated learning, Graph neural network, Large language modeling, Multimodal data fusion

## Abstract

Autism spectrum disorder (ASD) is a complex neurodevelopmental condition characterized by deficits in social communication and restricted behaviors. Traditional assessment and intervention methods rely heavily on subjective and time‐consuming approaches, which limit their clinical impact. Advances in artificial intelligence (AI) offer transformative opportunities for ASD research and practice. This narrative review proposes six AI‐driven strategies that address six core research challenges: uncovering causal mechanisms, modeling dynamic neurodevelopment, integrating multimodal data, individualized computational modeling, collaborative learning across institutions, and enhancing social training. We highlight the potential of causal inference to clarify gene‐environment interactions, spatio‐temporal graph neural networks to capture neurodevelopmental heterogeneity, and multimodal fusion for unified representation learning. Digital twin technologies enable personalized brain modeling and neuromodulation optimization, while social brain reverse engineering and federated learning frameworks support computational hypothesis generation and privacy‐preserving collaboration, respectively. Large language models further facilitate context‐aware social interventions. We also discuss key challenges—including data heterogeneity, interpretability, ethics, and clinical translation—and outline directions for building a more precise, human‐centered research paradigm. This review aims to move beyond incremental tool improvements toward reconstructing scientific paradigms, thereby accelerating the effective translation of AI innovations into clinical ASD applications.

## Graphical abstract

Six artificial intelligence strategies advance autism research from tool optimization to paradigm shift: causal modeling, spatiotemporal networks, multimodal integration, digital twins, social cognition mapping, collaborative learning, and context‐aware interventions for precision care.

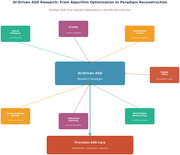

## INTRODUCTION

Autism spectrum disorder (ASD), a complicated neurological condition, has an impact on communication and social interactions. It also leads to constrained interests and compulsive actions.^1^ ASD has become more common in recent years. It affects about one in 31 young Americans.^2^ Approximately one in every 100 children worldwide has ASD.^3^, ^4^ This trend costs families a lot. Additionally, it places more strain on the healthcare system.

The complex and diverse etiology of ASD, together with its myriad symptoms and individual differences, makes the assessment and diagnosis of ASD continually challenging.^5^ Traditional diagnostic methods mostly rely on behavioral assessments and observational tools, which may be subjective and time‐consuming.^6^

Given these constraints, rapid advancements in artificial intelligence (AI) have provided unprecedented opportunities for early detection and tailored treatment of ASD. The significance of AI in medicine has increased significantly, leading to improvements in machine learning and deep learning for the prediction and early diagnosis of ASD.^7^, ^8^ While research encompasses AI's role in diagnosing and treating ASD, most studies are mostly algorithm‐focused and lack a systematic investigation into essential scientific questions concerning neurodevelopmental disorders^9^, ^10^ This perspective often overlooks the AI's ability to clarify causal pathways, inform intervention tactics, and enhance cross‐modal cognitive integration. This work provides a “task‐driven methodological pathway” to exceed the traditional method of “algorithm stacking.” It highlights six fundamental AI methodologies: causal modeling, graph neural networks (GNNs), multimodal fusion, digital twins (DTs), federated learning (FL), and large language models (LLMs). Each aligns with fundamental ASD research objectives: identifying causative pathways, modeling high‐dimensional dynamics, facilitating cross‐modal cognition, conducting customized brain modeling, fostering collaborative learning across data silos, and simulating social interactions. These strategies augment critical tasks while emphasizing their structural strengths and fundamental roles in theoretical development, mechanism design, and pathway interpretation.

**FIGURE 1:**
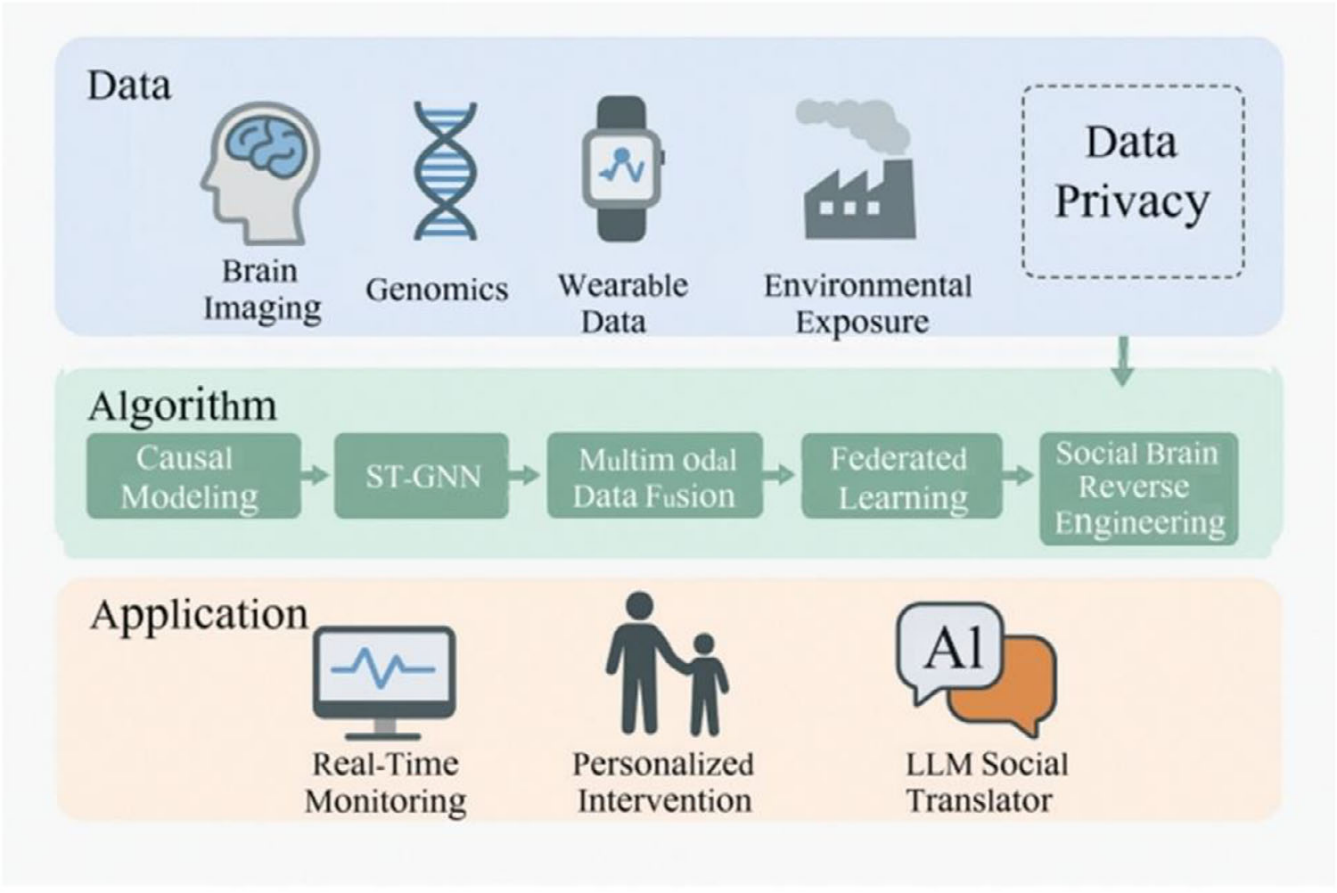
Flowchart of artificial intelligence‐driven autism spectrum disease research innovation frontiers. LLM, large language model; ST‐GNN, spatio‐temporal graph neural network.

This work establishes an AI‐driven research paradigm for complicated neurological illnesses by systematic investigation and integration of six pathways. It seeks to effect a paradigm shift from “optimizing tools” to “reconstructing scientific paradigms,” offering systematic assistance for clinical translation and systems modeling. Figure 1 presents a succinct diagram of the technological process.

This review constitutes a narrative rather than a predetermined systematic review, lacking comprehensive search strategies or evaluation of study quality. To ensure the inclusion of essential studies, several searches were conducted using PubMed and Google throughout the last 5 years. We integrated ASD‐related terminology (e.g., ASD) with evaluation‐related terminology (e.g., diagnosis) and specific AI keywords (e.g., AI, DT, FL, causal inference, multimodal fusion, GNNs, spatio‐temporal GNNs [ST‐GNNs], social robot, and LLMs) for a thorough search. Subsequent to deduplication, we swiftly evaluated titles and abstracts to eliminate irrelevant papers or those deficient in technical details. The complete texts of pertinent studies were examined and classified into six primary themes. Furthermore, we scrutinized the reference lists and utilized Google's “related documents” to enhance the search.

## FORMALIZING ETIOLOGICAL DYNAMICS: FROM CAUSAL INFERENCE TO ST GRAPH REASONING

In ASD research, pinpointing potential risk factors and intervening in their progression is a crucial yet formidable challenge. Many studies depend on cross‐sectional, longitudinal, or retrospective observational data. A longstanding dependence on correlation analysis has been employed to investigate risk variables, including the associations between maternal environmental exposures during pregnancy, early developmental indicators, and subsequent behavioral consequences.^11^ Nonetheless, correlation‐based approaches encounter difficulties in differentiating authentic causal correlations from superficial associations influenced by confounding variables, therefore constraining the use of study findings in clinical practice.^12^ Some research indicates strong connections between maternal PM2.5 exposure levels and childhood ASD risk.^13^ Nevertheless, does reducing maternal PM2.5 exposure effectively decrease ASD incidence? Can targeted behavioral therapies effectively mitigate social interaction challenges in specific subtypes of ASD? These inquiries frequently go unresolved due to insufficient causal evidence.^11^

This section elucidates the clinical significance of two technologies—causal inference and ST‐GNNs—by examining gene × environment (G × E) interactions, a pivotal aspect for comprehending individual variances in ASD pathogenesis research.^12^, ^14^ Conventional unimodal analysis often inadequately represents the dynamic regulation of biological processes, frequently resulting in suboptimal outcomes.^15^ These two technologies possess the capacity to surpass traditional low‐dimensional frameworks and encapsulate the ST progression of multi‐layered data.

### Causal inference as a formal mechanism for interpreting gene‐environment interactions

Causal inference is a statistical technique for detecting and modeling causal relationships between variables. In contrast to the conventional correlation analysis, its essence is in revealing intervention effects and causal sequences.^16^ Causal inference prioritizes the mechanisms of intervention and response between variables, in contrast to traditional statistical modeling, which stresses the strength of correlations among variables. It can address counterfactual inquiries, such as “How would the outcome differ if a specific intervention had not been executed?”^17^ This theoretical framework offers extensive applicability for causal inference in medical research. Especially in the lack of randomized controlled trials, it can facilitate the assessment of therapeutic benefits, the formulation of personalized solutions, and the enhancement of public health policies.^18^

In ASD research, the incorporation of causal inference methods enhances etiological exploration and intervention mechanism modeling while establishing a scientific basis for the effective translation of statistical findings into clinical decision support.^11^ The development of causal models helps identify “actionable risk factors,” such as specific environmental exposures (e.g., air pollution and heavy metals). These factors are implicated in disease risk through mechanisms like epigenetics or neurodevelopment. By intervening on these critical variables within designated time frames, we may potentially modify the developmental trajectories of ASD. A case‐control study conducted by Magen‐Molho et al.^19^ thoroughly evaluated the causal association between maternal exposure to PM2.5–10 and the likelihood of ASD. Findings indicated that genetically predicted PM2.5–10 exposure markedly elevated the incidence of ASD (odds ratio 1.08, 95% confidence interval [CI] 1.01–1.15), offering molecular epidemiological evidence for the causative relationship between “increased PM2.5 and ASD risk.” This also offers evidence‐based justification for enhancing prenatal air quality to diminish the prevalence of ASD.

Moreover, patients with ASD demonstrate considerable subtype distributions and individual differences, whereby a singular treatment protocol may be successful for certain individuals but ineffective for others.^20^ Causal inference methodologies—particularly individual treatment effect estimate and heterogeneous treatment Effect modeling—can elucidate “which individuals exhibit the greatest sensitivity to specific interventions,” hence facilitating the execution of stratified precision interventions. The Causal forest method introduced by Wager et al. facilitates the assessment of customized treatment effects in high‐dimensional data and has been utilized to evaluate the diverse outcomes of ASD therapies.^21^ The counterfactual regression model, created by Shalit et al., has been employed to forecast response discrepancies to therapies among patients with various ASD subtypes.^22^ Causal inference approaches offer a vital intervention‐oriented perspective for ASD research. Beyond basic correlation analysis, it aids in identifying causal risk variables that can be effective targets for prevention or intervention, along with appropriate intervention tactics for particular individuals or groupings. This method has been effectively utilized in various medical disciplines. In cardiovascular epidemiology, causal inference has established that reducing low‐density lipoprotein cholesterol decreases the occurrence of heart disease.^23^ Causal inference in public health clarified the relationship between smoking and lung cancer, therefore informing successful preventative strategies.^24^ These achievements highlight the necessity of employing stringent causal reasoning in ASD to improve the efficacy of early interventions and policy formulation.

### ST‐GNNs for modeling neurodevelopmental heterogeneity

Causal inference elucidates directional links between genes and environmental exposures; nevertheless, comprehending the temporal dynamics of ASD manifestations necessitates distinct modeling frameworks. These frameworks examine interactions among neural networks, behavioral patterns, and ecological environments. GNNs are deep learning architectures tailored for graph‐structured data, wherein items are denoted as nodes and relationships as edges.^25^ GNNs acquire intricate patterns through the dissemination of information along these connections.^26^

Tang et al.^27^ investigated dynamic functional connectivity and employed a GNN‐LSTM fusion model to enhance the diagnosis of ASD. They depicted brain connections at each temporal instance as a graph, inputting these time series graphs into a GNN‐LSTM. This method markedly improved the precision of classifying ASD compared to typical development by utilizing alterations in brain network architectures. It did not depend exclusively on static connectivity. Chen et al.^28^ proposed an adversarial learning‐based node‐edge graph attention network (AL‐NEGAT) that models both node and edge features from structural and functional magnetic resonance imaging (MRI) data to leverage complementary brain information. In AL‐NEGAT, the node‐edge graph attention mechanism preserves this information in subject‐specific weighted adjacency matrices, achieving 74.7% classification accuracy in distinguishing ASD from typically developing controls and outperforming several state‐of‐the‐art methods.

A separate study develops heterogeneous group graphs utilizing functional MRI (fMRI) and phenotypic data. It employs a multi‐layer heterogeneous graph convolutional attention network, a neural network engineered for analyzing intricate graphs with varied node and edge types, to derive graph‐based properties from the generated graphs. The features are then processed by a multi‐layer perceptron, a conventional neural network classifier, with a peak classification accuracy of 82.9% for ASD.^29^ In air quality forecasting, Lin et al.^30^ created a model that integrates multi‐source geographical data, including urban sensors, with temporal trends to estimate PM2.5 concentrations. Xiao et al.^31^ employed a dual‐path dynamic graph model, a neural network that identifies patterns in both spatial and temporal dimensions, to forecast air quality by modeling geographic proximity influences and temporal trends.

These studies illustrate the potential of sophisticated graph‐based and spatiotemporal models for examining the impact of environmental factors on the emergence and development of ASD throughout time and geography. However, restricted sample sizes in ASD hinder the strong validation of causal links. ST‐GNNs address the challenges of dynamic, developing graphs and facilitate transfer learning.^32^, ^33^, ^34^ The model is first pre‐trained on large‐scale public ST datasets using a self‐supervised objective to capture generic structural–temporal patterns.^35^, ^36^ A smaller ASD dataset, such as a birth cohort with environmental exposure measures, was utilized to refine the model for testing critical events, such as the trend of blood lead levels exceeding a specific threshold prior to alterations in ASD‐related biomarkers.^37^, ^38^ This two‐step training, a form of self‐supervised learning, can enhance the model's stability and generalization capacity when target data is restricted. Allow ST‐GNNs to model the alterations in interactions among various brain regions throughout development, or the evolution of gene‐environment linkages over time, thereby offering novel insights into elucidating the heterogeneity and progression of ASD symptoms. Neuroscience applications, like the modeling of Alzheimer's disease propagation and seizure prediction from electroencephalograms, underscore the extensive utility of ST‐GNNs and indicate their substantial potential influence on ASD research.^39^, ^40^

In conclusion, ST‐GNNs surpass simple prediction instruments, establishing a computational framework that amalgamates hypothesis generation, dynamic process simulation, and structural representation.^41^ They facilitate scalable investigation of ASD heterogeneity, aid in biomarker identification, and offer a theoretical framework for time‐sensitive, physiologically informed precision intervention design.

## SEMANTIC FUSION OF MULTIMODAL SIGNALS: TOWARDS CROSS‐SCALE REPRESENTATION LEARNING IN ASD

In AI‐driven ASD research, the advancement of data collection and integration techniques is essential to surmount conventional research constraints.^42^ Conventional data collection techniques predominantly depend on questionnaires or fixed evaluations, hindering researchers' ability to capture the continuous and dynamic multimodal signals of humans in natural environments, particularly regarding spatial behavior patterns.^43^, ^44^ This results in researchers lacking the requisite precision and comprehensiveness in evaluating intricate behavioral trajectories, with evident constraints in processing extensive data and fulfilling real‐time demands.^45^, ^46^ To tackle these problems, multimodal data integration techniques have become essential in AI‐driven ASD research. Multimodal data fusion is a hierarchical information processing technology. Integrating diverse data types into a cohesive model enhances the capacity of the system to recognize and comprehend complex situations.^47^ In neuroscience, multimodal data fusion can elucidate links across several dimensions, such as between intricate brain structures and their overarching functions, through the simultaneous analysis of brain imaging, genetic, and behavioral data.^48^, ^49^ This procedure elucidates the biological mechanisms underlying disease. Research on ASD has utilized multimodal brain imaging data to categorize the neuroanatomy of individuals with ASD, identifying three anatomical subtypes: ASD‐I, ASD‐II, and ASD‐III.^50^ These subtypes are then associated with behavioral phenotypes (observable behavioral patterns) and resting‐state (rs) functional connectivity patterns (patterns of brain activity during rest). Qi et al.^51^ examined covariate patterns in ASD by combining structural MRI, which depicts brain anatomy, fMRI, which illustrates brain activity, and other imaging techniques, uncovering both shared and unique neural network features within ASD subgroups. Multimodal data fusion approaches can merge with robotic and multisensor systems that amalgamate several sensing technologies.^52^ Robots designed for training can aid individuals with ASD in social skills development, shown as social assistance robots.^53^ Studies indicate that children with ASD exhibit a more favorable response to spoken directives from robots compared to direct instruction from therapists.^54^ The robots also precisely identify emotions displayed on synthetic faces.

Moreover, multimodal fusion approaches can simultaneously connect the internal brain activity of ASD with physiological alterations in the autonomic nerve system.^55^ Recent studies have derived behavioral features, including gaze direction and facial expression, from video data, and temporal indicators, such as heart rate variability, from physiological data.^56^ They conducted feature‐level (early) or decision‐level (late) fusion following temporal alignment to collaboratively model the relationship between behavior and physiological response. Utilizing a ST convolution and multi‐task learning framework, the current research has concurrently enhanced the diagnostic and intervention efficacy metrics in the integrated prediction of video, audio, and physiological signals, thereby markedly augmenting the model's robustness and generalization capacity.^57^ This transpires during the simultaneous prediction of visual, audio, and physiological inputs, significantly improving model resilience and generalization. In neurology, brain imaging has been integrated with multimodal data, including clinical and cognitive scales, for the assessment and forecasting of cognitive impairment associated with cerebrovascular illness.^58^ Certain models demonstrated performance comparable to expert clinical judgment in independent validation and were capable of identifying minor cross‐modal correlation patterns.

Multimodal fusion technology continues to encounter problems, including complex data synchronization, cross‐modal feature alignment, and inadequate interpretability.^59^ By leveraging successful experiences from other domains and persistently enhancing fusion techniques, ASD researchers can advance towards a thorough comprehension of this barrier, synthesizing diverse insights from biology to behavior into a coherent and predictable model.

## NEUROBEHAVIORAL DT: A VIRTUAL LABORATORY FOR ASD MECHANISM MODELING AND PERSONALIZED INTERVENTION OPTIMIZATION

### Neurobehavioral DT as a virtual laboratory for ASD mechanisms

Research indicates that the mechanisms of ASD are diverse and intricately linked to behavior, rendering single‐scale analyses insufficient to account for all patient variations.^60^, ^61^ The variation in ASD arises from intricate connections among genes, synapses, and behaviors.^60^, ^62^ Mutations in genes such as the ASD‐related *SHANK3* can disrupt synaptic scaffolding proteins.^62^ This disruption leads to dendritic spine abnormalities and compromises synaptic signal transduction, which alters neural network activity. As a result, these changes can manifest as behavioral anomalies associated with ASD, particularly in social interaction and sensory processing. Consequently, to diagnose and address ASD with more precision, it is essential to incorporate multi‐scale modeling of data, including molecular, structural brain area, and behavioral information, to study intricate pathogenic causes.^63^

DT technology facilitates the development of the aforementioned cross‐scale model and offers a novel approach for investigating the neurological mechanisms and intervention strategies for personalized ASD.^64^, ^65^ DT was initially employed in engineering manufacturing, denoting the real‐time simulation and analysis of physical systems through high‐precision virtual models.^66^, ^67^ Medical DT is not synonymous with conventional models or “virtual patients”; rather, it is a computational system tailored for specific individuals and perpetually linked to their behavioral and physiological data.^68^ It is capable of conducting condition monitoring, predicting outcomes, and optimizing interventions within a well‐defined context. In medical applications, DTs amalgamate multi‐source data pertaining to diseases, including imaging, genetics, and patient records, to improve prediction, prevention, and treatment.^63^ DT can accurately reproduce specific anatomical structures and dynamically replicate physiological processes, including hemodynamics and metabolic control.^69^ In neuroscience, DTs act as virtual simulations of the brain, emulating its functions and abnormalities.^70^ In oncology, multidisciplinary teams have been utilized to amalgamate patient and tumor genetic data, clinical histories, and imaging findings.^71^, ^72^ This facilitates the simulation, analysis, and forecasting of cancer growth and treatment results in virtual settings. Furthermore, regarding social behavior, the technology can utilize wearable gadgets and computer vision to collect data on micro‐expressions and movement.^73^ It is programmed as a dynamic node to simulate and assess the individual's physiological and behavioral condition, anticipated to facilitate individualized treatments for ASD. In conclusion, by including multimodal data, DTs can create personalized virtual models applicable across diverse domains. In the future, should technology advance and clinical trials be conducted to develop a model appropriate for the diagnosis and treatment of ASD, the prediction, prevention, and management of ASD will substantially improve.

### DT‐based “intervention sandbox” for dynamic neuromodulation optimization

One concrete application of DT technology is the creation of a secure testing environment, or “sandbox,” in which candidate interventions can be simulated and optimized before implementation.^74^ In clinical practice, selecting appropriate interventions for individuals with ASD often still relies on repeated trial‐and‐error.^75^ The sandbox methodology, originally developed in the financial technology sector, denotes a virtual environment that is segregated from real‐world systems and used for the collaborative development and testing of innovative products, methods, and regulatory schemes.^76^ Translated into the neuromodulation context, a DT‐driven virtual “intervention sandbox” safely and efficiently evaluates multiple intervention strategies, thereby enabling systematic exploration of the neuromodulation parameter space and refinement of individualized treatment plans.^64^ Takahashi et al.^77^ employed DT concepts to create a real‐time electroencephalogram simulator that forecasts alterations in brain state and mimics pharmacological therapies, illustrating the practical implications of DT‐based sandboxes. Lynch et al.^78^ proposed “targeted functional network stimulation” in neuromodulation, a technique that enhances transcranial magnetic stimulation coil positioning based on individual neuroanatomy, demonstrating the efficacy of individualized digital environments. Likewise, Rashed et al.^79^ utilized deep learning for personalized head models to forecast brain electric field distributions during transcranial magnetic stimulation, reinforcing the principal assertion that DT technology distinctly facilitates the secure, systematic, and tailored optimization of ASD intervention strategies.

In summary, DT technology facilitates the systematic investigation of neuromodulation parameter spaces and the optimization of tailored treatment strategies by allowing the creation of virtual “intervention sandboxes.” This core competence provides novel, personalized strategies for therapies in neurodevelopmental disorders, including ASD.

## “SOCIAL BRAIN REVERSE ENGINEERING”: INFERRING ASD MECHANISMS FROM AI SOCIALITY

The predominant characteristic of ASD is impaired social interaction, typically exhibited as challenges in comprehending others’ ideas and emotions (i.e., a deficiency in “theory of mind”) and unusual social communication behaviors.^1^ The concept of the “social brain” provides a neurobiological framework for understanding the social cognitive impairments seen in ASD. Subsequent research following Brothers’ proposal reveals that the prefrontal cortex, amygdala, and superior temporal gyrus are crucial for understanding others’ emotions, intentions, and relational dynamics.^80^, ^81^, ^82^, ^83^ These regions collaborate to facilitate effective social responses, understanding of behaviors, and adaptation to diverse situations. Nonetheless, a definitive brain mechanism that comprehensively elucidates the social difficulties experienced by individuals with ASD remains elusive.

In recent years, AI has exhibited “social” competencies while doing various tasks. Advanced robots or conversational agents have the ability to recognize faces and analyze emotions from verbal signals.^84^ They possess the ability to generate human‐like speech and can collaborate with individuals in gaming endeavors. This raises a question: Can we employ AI systems as tools to understand the human social brain, especially with ASD? Comprehending the mechanisms by which AI algorithms produce social behaviors may clarify the social cognitive impairments identified in ASD. This approach is termed “social brain reverse engineering.”^85^

Our objective is to transform “social brain reverse engineering” into a novel approach for understanding the social difficulties related to ASD. This is achieved by integrating multimodal dynamic modeling with cross‐species cognitive frameworks. “Social brain reverse engineering” involves analyzing social behaviors and the internal computational structures of artificial agents, such as social robots, to understand their interactions with humans.^85^ This enables us to infer the unconventional systems regulating social cognition in individuals with ASD. This paradigm perceives AI models as enablers of controllable social behavior and cognitive mapping frameworks. By contrasting their “ideal response paths” with the actual responses of individuals with ASD, we can provide computable explanations for cognitive anomalies, including deficits in theory of mind.

In this setting, researchers employ adversarial social training techniques. They first outline the typical social characteristics of ASD, such as stereotyped responses and reduced eye contact, as target behaviors.^1^ They subsequently train social robots using reinforcement learning methodologies. In human‐robot interactions, the robot continually adjusts its methods. It achieves this by integrating multimodal data, including facial expressions, eye‐tracking patterns, and vocal affect.^84^, ^86^ The robot employs feedback optimization to construct a computational model of the “social brain.” By obtaining multimodal data, researchers can develop customized machine learning frameworks for individuals. This enables robots to sense the emotional atmosphere during real‐time interactions and promptly modify their behavioral decisions.^87^, ^88^

Research has implemented this approach through emotion‐aware robots to capture children's facial expressions and voice emotions in real time. These robots swiftly detect adverse emotions for assistance. This technique promotes beneficial emotional interactions among children and aids in their emotional management. And this method can enhance children's good emotional interactions and assist them in regulating their emotions. In the real‐time interactive setting, the precision of facial expression detection is 85%, while the accuracy of voice emotion analysis is 83%, demonstrating effective functionality.^89^ This approach can also allow the robot to optimize its internal “social brain” model in a reverse manner within a dynamic social environment, thereby identifying critical factors and mechanisms that influence social interaction.^90^ By constructing an ideal social path with AI, an AI agent gains self‐social cognitive modeling ability. It can clearly identify where individuals with ASD lack or deviate in key cognitive areas, such as emotion recognition and intention inference.^91^, ^92^

The research may also incorporate the macaque ASD model with the robot's cognitive framework. This methodology seeks to establish a cross‐species “cognitive mapping bridge.” Macaques are monkeys that share a close evolutionary relationship with humans; their ASD models exhibit neurological and behavioral characteristics akin to those of human ASD.^93^ Incorporating these behavioral and neurological data into social robot control systems improves behavioral biomimicry. It also confirms the progression from researching ASD causes to virtual simulation and enhancing intervention options.

The objective of “social brain reverse engineering” is to construct a robot that mimics human interaction while simultaneously elucidating the fundamental principles of human social behavior. It also seeks to create a measurable and substantiated cognitive framework. This paradigm converts conventional psychological ideas, such as “theory of mind,” into verifiable computational models. It can subsequently provide more tailored and reliable strategies for social treatments in ASD.

## FL AND CROSS‐CENTER COLLABORATIVE MODELING: AN INTELLIGENT DATA COLLABORATION PARADIGM FOR ASD RESEARCH

Healthcare systems encounter increasing conflict between data silos and privacy issues. Institutions collect extensive high‐dimensional data but have difficulties in sharing information between enterprises due to privacy and security laws.^94^ This insular ecology obstructs the integration and dissemination of cross‐regional information. FL mitigates this issue by facilitating data exchange while preserving privacy.^95^ It facilitates model training across users' devices, maintaining data locality while enabling collaborative model development.^96^ A global FL model, EXAM, which encompasses nearly 20 hospitals, forecasted oxygen requirements in coronavirus disease 2019 patients with an area under the curve (AUC) over 0.92 and demonstrated extensive applicability.^97^ Multicenter clinical research utilizing FL for the detection of cerebral bleeding attained an AUC of 0.9487 (95% CI 0.9471–0.9503), whilst a non‐FL model exhibited an AUC of 0.9753 (95% CI 0.9742–0.9764).^98^ These findings indicate that FL may acquire knowledge from heterogeneous populations and generate precise, generalizable models without the need for data centralization.

FL in ASD research encounters obstacles, including the equilibrium between consensus and individual distinctiveness. We present a heterogeneous model fusion approach employing a dual‐track strategy that integrates knowledge distillation and mixture‐of‐expert architectures.

Knowledge distillation is a technique that, similar to conventional FL, mitigates privacy issues and computational expenses.^98^ It also enables diverse client models, which may differ in size, precision, or architecture, to train collaboratively.^99^ In a federated environment, knowledge distillation applies to any model type. For example, one institution may use a large CNN, while another uses a small random forest. They can extract their models’ knowledge, like prediction distributions on public datasets, into a common soft output format. Finally, aggregation is performed on the server side.^100^, ^101^

The mixture‐of‐experts technology facilitates the development of models that are more appropriately tailored to local datasets, while enabling the collaboration of several expert models.^102^, ^103^ The technique employs a gating mechanism to allocate dynamic weights to spliced multimodal inputs. These inputs may comprise photographs, text, and genetic information. The system directs them to designated model specialists, including CNN, transformer, or GNN. This accomplishes input‐dependent, heterogeneous expert activation and integration. The process enhances flexibility for diverse multimodal data. It also improves performance in other downstream operations.^104^, ^105^

In recent years, FL has been utilized to train models on multi‐hospital cancer datasets and for distributed MRI and computed tomography analysis.^106^, ^107^ In ASD research, FL has been utilized to analyze functional connectivity derived from rs‐fMRI data across multiple sites, enabling joint modeling while preserving privacy without sharing raw data.^108^ FL assists researchers in surmounting the constraints of limited sample sizes and singular study facilities. This facilitates the earlier detection of ASD in bigger, more heterogeneous groups, thus enhancing model generalizability.^109^, ^110^ The integration of these approaches allows the FL framework to provide privacy‐preserving modeling of multicenter medical data while balancing generalization and the representation of individual differences. This establishes a novel framework for cross‐institutional intelligent modeling of extremely varied disorders such as ASD.^108^

## LLM‐DRIVEN SOCIAL REASONING: EXPANDING CONTEXTUAL MODELING AND EMPATHY GENERATION CAPABILITIES IN ASD RESEARCH

LLMs represent a nascent medium for communication, which exhibits promise as “cognitive intermediaries” between individuals with ASD and neurotypical populations.^111^ LLMs, such as GPT‐3 and GPT‐4, exhibit remarkable human‐like language comprehension and generation abilities.^112^, ^113^ These models are trained on massive amounts of data and are capable of answering questions, conducting conversations, and even performing certain inferences.^112^ Studies indicate that brief training with LLM leads to substantial enhancements in empathy among persons with ASD. These advancements enhance social discourse, providing novel methods to surmount obstacles associated with social communication in ASD.^114^

### Semantic translation of nonverbal signals: An interpretable modeling approach for cross‐modal social information

Individuals with ASD frequently exhibit nonverbal communication that markedly differs from that of neurotypical individuals. This encompasses diminished facial expressions, reduced eye contact, and atypical body posture^115^, ^116^, ^117^ The disparities frequently lead neurotypical individuals to misinterpret the social intentions of those with ASD, thereby obstructing communication. Research indicates that these disparities are not merely the result of unilateral impairments, but rather a ‘dual empathy problem’—fundamental variations in the manner in which neurodiverse groups absorb social information.^118^ Multimodal LLM systems, utilizing computer vision and linguistic techniques, can identify and comprehend nonverbal behaviors in individuals with ASD.^119^ For example, a smartphone camera can identify children's abnormal behaviors, such as eye avoidance and abnormal gestures. These visual features and context data can then be received through LLM (such as informing children's environment) to generate explanations and suggestions for the children's current social status.^120^, ^121^ Providing real‐time feedback to parents or educators enhances their comprehension and responsiveness to the child.

Evidence is beginning to corroborate this methodology. For example, the multimodal model transforms visual features into behavioral labels, such as “object operation” and “head self‐injury.”^122^ It then generates text descriptions of these patterns using the language‐reasoning ability of an LLM, such as GPT‐4. This significantly improves recognition accuracy and interpretability for restricted and repetitive behaviors in children with ASD. This technology‐based ‘behavioral translation’ helps mitigate misconceptions and enhance social interactions with youngsters diagnosed with ASD. Nevertheless, the majority of contemporary research concentrates on direct consequences, such as system correctness and clarity, and has yet to illustrate its influence on enhancing elements like peer connections. Future research with a rigorous design is necessary to validate these findings.^114^, ^123^

### Flexible construction of social rules: From structured scripts to dynamic strategy generation

Conventional ASD social training frequently depends on inflexible guidelines and predetermined scripts. This renders individuals with ASD incapable of maneuvering through intricate and evolving social norms in daily interactions. LLMs can facilitate the surmounting of this obstacle by generating adaptable social training environments.^124^ The utilization of models such as GPT‐3 and ChatGPT for the creation of social narratives for individuals with ASD represents an emerging area of research.^125^ Studies demonstrate that, in contrast to conventional limited scenario training, GPT‐series models are capable of producing a variety of social contexts. This broadens the range of training situations.^126^ LLMs provide significant flexibility in social rule learning by establishing a spectrum of complexity, ranging from highly structured to entirely natural scenarios. Recent research indicates that agent‐based communities influenced by LLMs demonstrate the spontaneous formation of social norms.^127^, ^128^ This discovery further corroborates the capacity of these models to dynamically formulate and adjust social regulations.

Lan et al.^129^ used the public health driver transformer model to integrate multimodal data (such as text, audio, and facial cues) to provide real‐time social context interpretation and adaptive feedback for ASD. This approach enables a more natural and engaging learning experience to improve the social skills of children with ASD. Moreover, research indicates that LLMs can markedly enhance empathy expression and social confidence in teenagers and adults with ASD in brief periods.^114^ This establishes an empirical basis for the wider use of their findings in social training for ASD patients. Although these studies illustrate the potential of LLMs for immediate empowerment, their transferability across languages and cultures, long‐term efficacy, and overall advantages in practical intervention frameworks still necessitate evaluation. Validation must be achieved through multicenter, pre‐registered randomized controlled trials.

LLMs are transitioning ASD social training from “rule‐based instruction” to “contextual co‐creation.” They have distinctive capabilities in semantic generation and scenario adaption.^130^ Research suggests that these AI systems ought to be seen as “empowering tools” that augment, rather than supplant, healthcare and educational endeavors.^114^ They should be utilized in conjunction with human experts. At present, these models encounter difficulties in transfer learning, managing semantic bias, and addressing neurodiversity.^111^ Future enhancement of LLMs necessitates the use of personalized behavioral feedback systems alongside elucidative modeling techniques.

## CHALLENGES AND OPPORTUNITIES

AI presents both opportunities and obstacles in the diagnosis, intervention, and treatment of ASD.^131^ These encompass not only technology innovation but also data integration, privacy ethics, and clinical translation. In this dynamic domain, researchers must confront these obstacles through methodical technology innovation and theoretical synthesis to elevate ASD research to greater levels of comprehension. Following prior discussions on AI development concerns, we now give the existing challenges and potential in a more succinct fashion for the reader's convenience, as illustrated in Table 1.

**TABLE 1:** Opportunities and challenges in artificial intelligence (AI)‐driven autism spectrum disorder (ASD) research

Challenge area	Opportunities and solutions
**Data heterogeneity and multimodal fusion**	Formulate sophisticated multimodal data fusion techniques to concurrently synchronize temporal and spatial attributes from diverse data sources, hence improving the precision of integrated analysis. Present self‐supervised learning. Utilize self‐supervised pre‐training to identify commonalities in extensive spatiotemporal data, subsequently fine‐tuning on restricted ASD birth cohorts to enhance model stability and generalization efficacy.^132^
**Data privacy and data silos**	Implement distributed training frameworks, such as federated learning, to provide multi‐center collaboration without necessitating the exchange of raw data.^95^ Utilize stringent methods, including differential privacy and impermeable encryption, to guarantee data confidentiality throughout parameter transmission.^133^ Establish explicit ethical principles and legal frameworks for data exchange, with standards similar to those in the United States. The Health Insurance Portability and Accountability Act (HIPAA) and the European Union (EU) General Data Protection Regulation (GDPR) can significantly improve inter‐institutional trust.^134^
**Insufficient model interpretability**	Incorporate explainable AI technologies, including attention processes and model visualization tools, to improve transparency and comprehension.^10^ Illustrating attention weights and causal links enhances transparency in data utilization, hence facilitating the synchronization of AI findings with clinical workflows. Promote the development of simplified, interpretable models for diagnostic assistance to aid physicians in understanding and validating AI results.
**Clinical translation and standardization barriers**	The present algorithm's performance is inadequate for clinical use; a transparent and verifiable regulatory framework is also essential. In the United States, AI medical devices are required to be registered and regulated by the Food and Drug Administration (FDA) during their operational lifespan.^135^ Upon software upgrades, a predetermined protocol must be adhered to for modifications to regulate updates, including retraining, establishing new thresholds, and evaluating performance, so as to ensure the devices remain secure and efficient.^136^, ^137^ The EU's AI Act classifies medical AI as high‐risk and governs it alongside current medical device restrictions.^138^ This indicates that clinical testing and post‐release evaluations persist; however, additional mandates for data integrity, human supervision, transparency, and risk management have been established. Official documents indicate that the AI Act operates alongside current medical device rules, requiring makers to maintain records for both regulatory frameworks.^139^ Studies indicate that AI technologies designed to assist in autism diagnosis can achieve accuracy comparable to that of experienced physicians in clinical evaluations performed across multiple centers.^140^, ^141^ Consequently, these devices have obtained FDA approval for utilization in children aged 18 to 72 months.
**Economic costs and data sharing issues**	Total Cost of Ownership (TCO) includes significant expenditures such as initial data management and validation, security improvements, employee training, process modifications, and continuous assessment and retraining.^135^ To demonstrate sustainability, payment mechanisms must be associated with cost analyses, including delays, referrals, and cost per case.^142^, ^143^ The FAIR Principles (Findable, Accessible, Interoperable, and Reusable) offer explicit recommendations that promote data sharing and improve model training and reuse in the context of open science.^144^
**Cross‐border technical feasibility and variability issues**	Multiple critical factors—health insurance reimbursement mechanisms, staffing, data governance, and international transfer (e.g., under GDPR), technological risks across diverse languages and cultures, and infrastructural disparities—significantly influence the pace of new technology adoption.^145^ Industry associations caution that the concurrent or separate implementation of the AI Act and Medical Device Regulation (MDR) may impede patient access to safe and effective instruments. To mitigate these delays, consistent retesting, limited updates, and performance evaluations should be integral to continuous updates in accordance with the United States.^138^ Planned Change Control Plan and Good Machine Learning Practice; this ensures that modifications are monitored and quality is upheld.^146^ Employing standard data principles (FAIR principles) facilitates sharing and reuse, while testing technology across multiple hospitals assesses its consistent efficacy.^144^, ^147^ Ultimately, assess the sustainability of solutions by analyzing the structure of payments.
**Fairness and ethical considerations**	To identify and mitigate potential biases, include varied population data during model construction, providing equitable benefits for individuals with ASD across genders, races, and socioeconomic statuses.^148^ Furthermore, implement comprehensive informed consent and ethical review processes to thoroughly honor patient and family wishes prior to AI‐assisted therapies.^149^ Additionally, incorporate interdisciplinary collaboration with specialists in ethics and sociology to collectively monitor the societal implications of AI applications, ultimately bolstering public trust in AI.

Abbreviations: AI, artificial intelligence; ASD, autism spectrum disorder.

## FUTURE DEVELOPMENT DIRECTIONS

Future AI‐driven ASD research has potential for significant paradigm shifts. Initially, longitudinal trajectory tracking will be crucial in comprehending the dynamic evolution of ASD. By including high‐frequency physiological inputs, environmental exposure omics, and single‐cell gene expression, AI models may identify nuanced alterations in individual development in real‐time. This facilitates continuous modification from risk assessment to intervention enhancement. As longitudinal data is collected, researchers can formulate individualized developmental trajectories. This facilitates the early identification of abnormalities and the determination of suitable intervention periods. The advancement of multimodal DTs will facilitate the emergence of novel platforms in ASD research and intervention evaluation. Integrating data from single‐cell RNA sequencing, wearable physiological devices, and environmental sensors enables virtual models to simulate various intervention options without risk. When integrated with closed‐loop systems enhanced by reinforcement learning, DTs can precisely forecast outcomes and identify the most efficacious therapies. This offers technological assistance for prompt, individualized, and immediate interventions. Simultaneously, privacy‐preserving and fairness‐oriented FL will underpin cross‐center collaboration. Confronted with data silos and privacy regulations, forthcoming ASD research will employ privacy protection, safe multiparty computation, and heterogeneous model fusion. This allows medical institutions to collaboratively develop models that cater to both specific and general requirements, while preserving the confidentiality of raw data. This partnership improves robustness and clinical significance while mitigating bias that could impact disadvantaged populations. Ultimately, explainable AI will improve model transparency and its application in healthcare settings.^150^ Through the application of attention processes, causal modeling, and visualization, forthcoming AI models will go beyond mere prediction. They will provide causal explanations related to neuronal, chemical, and physiological signals. This will assist doctors in comprehending decisions and fostering trust in AI‐enhanced patient and family care.

Through the ongoing enhancement of technologies for longitudinal monitoring, online assessment, secure collaboration, and accessible AI, AI presents a genuine promise for substantially increasing research and care in ASD. This modification will shift from static evaluations to continuous monitoring and from generic interventions to more precise modifications, with the objective of providing support that is more integrated, accurate, and individualized.

## CONCLUSION

The swift progression of AI technology is transforming the early identification of ASD and the customization of targeted interventions. AI systems currently amalgamate neural activity signals, behavioral observations, and comprehensive biological data into a cohesive analytical framework. This allows AI to recognize intricate patterns and clarify fundamental mechanisms, while maintaining the original time and structure of the data. This signifies a shift from mere diagnosis to a focus on comprehending the mechanisms of ASD and anticipating responses to various treatments. Significant issues persist, including safeguarding privacy, ensuring the transparency and comprehensibility of AI models, and facilitating the transfer of models between institutions. Nevertheless, innovative methodologies present prospective resolutions. FL entails training AI models on locally stored data, so ensuring that sensitive information remains at its original location. Differential privacy introduces noise to data to safeguard identities, whereas GNNs are computational models that examine relationships within data, now enhanced with increased transparency to elucidate decision‐making processes. In the future, AI may integrate knowledge accumulated over years from various sources and employ multiple analytical tools, including those that interpret and analyze social behavior data. This could propel ASD therapies into a phase of enhanced personalization, continuous feedback, and adaptable modifications. These tools will link clinical environments with home care, enhancing the quality of life and support for individuals with ASD and their families. By integrating advancements from neuroscience, computer science, and medicine, AI will unify theory and practice. This partnership will advance future ASD treatment.

## CONFLICT OF INTEREST

The authors declare no conflict of interest.
